# The Interim Report of the Committee of Inquiry

**Published:** 1916-06-17

**Authors:** 


### NATIONAL INSURANCE ACT DEVELOPMENTS.

### The Interim Report of the Committee of Inquiry.

The Interim Report of the Departmental Committee, appointed by the Treasury to inquire into national health insurance matters, which has just been published, is, perhaps, the most interesting and illuminating Report which has yet been issued relating to the great scheme of insurance for the prevention and cure of sickness which was inaugurated by the National Insurance Act of 1911. It is, moreover, of great importance as indicating that in the near future considerable alterations will have to be made in the structure of the original Act.

The Report shows quite clearly that there was a real and urgent need for such an inquiry, notwithstanding the fact that for some months prior to the appointment of the Committee the Government had resisted all endeavours to secure the investigation of the administration of the Act with a view to putting its finance upon a sound basis.

One section only of the matter referred to the Committee—namely, the general scheme of finance upon which the whole superstructure of the insurance system is based—is dealt with in the present Report. In due course there is to be a further Report, dealing with the practical administrative reforms in matters of detail which should now be made as the result of the experience already gained. As the Committee express their anxiety to proceed expeditiously with the second part of their inquiry, there is reason to expect that their final Report will not be unduly delayed. In the meantime it may be observed that the object desired by the Committee in presenting an interim Report was to afford a reasonable time for the consideration of their proposals and for the preparation of any legislation arising therefrom which the Government may see fit to introduce at an early date.

### Fresh Legislation Proved to be Necessary.

That it will be necessary for legislation to be introduced is immediately evident upon carefully reading the Report, for it is first of all made clear that in certain vital respects the present financial basis is unsatisfactory. In fact, one might almost use a stronger expression and say, at least in regard to the provision for married women's benefits, that the present finance is unsound.

In face of the evidence placed on record by the Committee, the Government cannot allow the continuance of the present system, and the proposals for its rectification put forward by the Committee are of such a character as to make amending legislation inevitable.

The reasons adduced by the Committee for the failure of the original financial basis may be summed up by stating, that whereas the actuaries were asked to advise the Government on the basis of a flat rate of contribution applicable to the country as a whole, the administrative plan adopted allowed segregation of different types of insured persons, with the result that there are necessary departures from the standard experience. Thus, for instance, the Committee point out that as regards rural and agricultural societies the provision made under the Act exceeded that which would have been held to be necessary by competent authorities, on the basis of Friendly Society experience, by as much as 40 per cent. On the other hand, it is also pointed out that, in spite of the exclusion of industrial accidents, the provision made for persons engaged in unhealthy occupations fell considerably short of the actual requirements.

The test of the present system as a whole—not as applied to individual societies—is very clearly set forth in two simple tables which show that, as regards the benefits, there is very little difference between the actual cost and that expected in the case of men. For women's benefits, however, it is clear that the actual cost has largely exceeded the provision that was made for it.

There was a decrease in the actual cost in 1915 as compared with 1914 which is attributed to the abnormal industrial conditions due to the war, and it is not to be anticipated that the improvement shown would be other than temporary.

### Deficiencies on Valuation Foreshadowed.

Consideration of the above figures, or others of a similar character, has led certain critics of the Act to state that the National Insurance scheme was becoming insolvent. The Committee state, however, that "a condition of bankruptcy in the strict sense of the term cannot arise in the case of approved societies, since the present law provides the means by which societies can regain a position of financial equilibrium by reducing benefits or increasing the contributions of their members. But it is clear that, bearing in mind the general scheme of the Acts, a number of societies would under the existing law inevitably find themselves in deficiency on valuation, probably in some instances to a serious extent."

It is this disclosure of deficiencies, and the consequent reduction of benefits, which is desired to be averted as far as possible.

It would be possible theoretically, as the Committee point out, to apply the surpluses, in whole or in part, of those societies which have shown favourable experience to liquidate the deficiencies of those societies which have not been so fortunate. If this system were adopted, however, individual societies would lose the valuable inducement to effective administration which the prospect of a surplus holds out. In consequence, the Committee do not recommend any such plan.

Seeing that the terms of reference restricted the Committee to the consideration of proposals within the limits of the present contributions and benefits, and that further Exchequer grants were also excluded, the Committee were precluded from making suggestions of the most obvious kind which would have involved the increase of contributions or the decrease of benefits. Their only alternative, therefore, was to turn to the sinking fund, the object of which is to liquidate the initial reserves which were set up as book entries at the commencement of the Act to enable all employed contributors coming into insurance at that time to be treated as though entering at age sixteen.

Under the present scheme, out of each weekly contribution a proportion—namely, one penny and five-ninths for men, and three-halfpence for women—is carried to the reserve account to provide interest on the book reserves and to convert these reserves ultimately into actual invested funds. Not until the book reserves have been actually converted into cash funds will it be possible for these substantial proportions of the contributions to be applied towards the provision of the additional benefits which are held out in expectancy under the Act. And not until the complete liquidation of the book reserves will the insured receive immediate benefit from the proportion of benefits paid by the State.

It follows, therefore, that any diminution of the rate at which the sinking fund accumulates must automatically defer the time when the additional benefits will become payable.

### Resort to the Sinking Fund—A Twenty-year Extension.

It will be remembered that according to the original estimate it was computed that it would take about fifteen years before the reserves would be liquidated. Already various encroachments upon the sinking fund have extended the term to twenty years. The further demands upon the sinking fund which the Committee now suggest will, if adopted, extend the term by an additional period estimated at six years, so that instead of the additional benefits being available in the year 1932, they will not emerge until 1938. It is, however, pointed out by the Committee that the result of their proposals should be brought under review in ten years' time, and in the calculation upon which the extension of six years is based they have assumed that their proposals will only be in operation for ten years. It must, however, be supposed that on the expiration of the period of ten years specified, it will be necessary to continue the scheme now put forward with little or no variation, and, if this should actually be necessary, the Committee state that the extension of the term before which the reserves will be liquidated will be not six years but twenty. Thus there is every prospect that it will not be until the year 1952 that the effect of taking the original entrants into insurance as though all were then aged sixteen years will be eliminated.

### The Proposals Justified.

The concluding paragraphs of the Report contain such an excellent piece of special pleading on behalf of the proposals made, and such a direct warning that it will not be possible to make further concessions or allowances, that we venture to give them almost in full.

"In the course of this Report," the Committee state, "we have endeavoured to show that the solution we have suggested to the problem submitted to us was in its main features pre-determined by the conditions of the case—that no alternative means were available to bring about the needed improvement in the financial position of societies to that of applying a portion of the sinking-fund contribution to the immediate purpose in hand—but we are of opinion that it may claim to be justified on its merits. Given the inadequacy of the present contributions in certain cases to sustain the present scale of benefits, it may reasonably be contended that the needs of the moment should take priority over the claims of the future. The assumptions on which the National Health Insurance scheme was based provided a balance of contribution-money, after making due provision for sickness and other claims, which was properly set aside to redeem the reserve value credits and be available for extended benefits after a given time. The actual experience of many societies since the Act of 1911 came into force shows that in the case of women this balance was considerably smaller than was supposed, and that in the case of both men and women a considerable draft upon it was required to overcome the inequalities of the flat-rate contribution. Hence it is natural that the original position should be reconsidered, and that, with a fuller knowledge of the situation, the provision for redeeming reserve values should be amended."

"But it is easy to prove that insured persons do not suffer injustice or hardship by reason of the proposals made in this Report. The same amount as has been hitherto devoted to redemption purposes will be available for the use and benefit of societies, the difference being that some part of it will be utilised earlier than the Act of 1911 contemplated, while the remainder will operate as a sinking fund which will redeem the reserv. values at a somewhat more distant date. Further, we have framed our proposals in such a manner as to leave open to reconsideration within a reasonable period those features in the scheme which in our view might after a time require modification in the light of the further experience then available. . . ."

### A Warning.

"We cannot, however, conclude the first part of our labours without emphasising the fact that the scheme we propose would absorb the resources at present available, and that while well-managed societies might reasonably expect under the scheme to be relieved from the necessity of reducing the benefits of their members on valuation, the scheme would not and is not intended to enable them to relax in any way their present standards of administration on the assumption that their resources, having been once supplemented, may be capable of an almost indefinite extension at some future date."

Space will not allow us to give in this issue the precise details of the scheme, but we hope to be able to indicate in an early issue the more important details of the manner in which the money diverted from the sinking fund is proposed to be employed in providing funds for immediate use.

